# Computational challenges and solutions: Prime number generation for enhanced data security

**DOI:** 10.1371/journal.pone.0311782

**Published:** 2024-11-15

**Authors:** Amal Ezz-Eldien, Mohamed Ezz, Amjad Alsirhani, Ayman Mohamed Mostafa, Abdullah Alomari, Faeiz Alserhani, Mohammed Mujib Alshahrani

**Affiliations:** 1 Department of Basic Science, Bilbeis Higher Institute for Engineering, Ministry of Higher Education, Bilbeis, Egypt; 2 Department of Computer Sciences, College of Computer and Information Sciences, Jouf University, Sakakah, Saudi Arabia; 3 Department of Information Systems, College of Computer and Information Sciences, Jouf University, Sakakah, Saudi Arabia; 4 Department of Computer Science, Al-Baha University, Albaha, Saudi Arabia; 5 Department of Computer Engineering & Networks, College of Computer and Information Sciences, Sakaka, Al Jouf, Saudi Arabia; 6 Department of Information Systems, College of Computing and Information Technology, University of Bisha, Bisha, Saudi Arabia; National University of Sciences and Technology, UNITED KINGDOM OF GREAT BRITAIN AND NORTHERN IRELAND

## Abstract

This paper addresses the computational methods and challenges associated with prime number generation, a critical component in encryption algorithms for ensuring data security. The generation of prime numbers efficiently is a critical challenge in various domains, including cryptography, number theory, and computer science. The quest to find more effective algorithms for prime number generation is driven by the increasing demand for secure communication and data storage and the need for efficient algorithms to solve complex mathematical problems. Our goal is to address this challenge by presenting two novel algorithms for generating prime numbers: one that generates primes up to a given limit and another that generates primes within a specified range. These innovative algorithms are founded on the formulas of odd-composed numbers, allowing them to achieve remarkable performance improvements compared to existing prime number generation algorithms. Our comprehensive experimental results reveal that our proposed algorithms outperform well-established prime number generation algorithms such as Miller-Rabin, Sieve of Atkin, Sieve of Eratosthenes, and Sieve of Sundaram regarding mean execution time. More notably, our algorithms exhibit the unique ability to provide prime numbers from range to range with a commendable performance. This substantial enhancement in performance and adaptability can significantly impact the effectiveness of various applications that depend on prime numbers, from cryptographic systems to distributed computing. By providing an efficient and flexible method for generating prime numbers, our proposed algorithms can develop more secure and reliable communication systems, enable faster computations in number theory, and support advanced computer science and mathematics research.

## 1. Introduction

Prime numbers have captivated mathematicians and computer scientists for centuries due to their unique properties and applications in various fields. They play a crucial role in cryptography, specifically in key generation for secure communication protocols such as the RSA cryptosystem. Additionally, prime numbers are fundamental in number theory and serve as building blocks for many complex mathematical problems, including but not limited to prime factorization, primality testing, and the distribution of primes. Efficiently generating prime numbers has been a long-standing challenge, and several algorithms have been proposed to tackle this problem. Some of the most widely known prime number generation algorithms include the Sieve of Eratosthenes, the Sieve of Sundaram, the Sieve of Atkin, and the Miller-Rabin primality test [1]. The fundamental use of primary numbers is through the encryption and decryption processes of different cryptographic algorithms in addition to the security of communications that generates pseudorandom sequence numbers that can be adapted in different security applications [2]. Due to their difficulty in factoring, prime numbers are frequently utilized in cryptography. The process of factoring involves identifying a number’s prime factors. For instance, prime numbers 2 and 3 can be factored into 12. A high number like 1024 is far more challenging to factor. Large numbers are challenging to factor, so prime numbers are so effective for encryption. The public key must be factored in order to decrypt a communication that has been encrypted. However, this extremely challenging process cannot be completed quickly.

However, each of these algorithms has its limitations and may only be suitable for some applications or when dealing with large ranges of numbers. The limitations can range from time and space complexity to the inability to efficiently handle a particular range of numbers [3,4]. In recent years, the increasing demand for efficient prime number generation has led to the development of new algorithms that can adapt to modern computers’ ever-growing computational power and memory capabilities. This has given rise to the need for better-performing algorithms designed to handle large ranges of numbers and can provide quick and accurate results.

This paper presents two novel algorithms that address the challenge of generating prime numbers up to a given limit and within a specified range. These algorithms are based on the formulas of odd-composed numbers, which allow them to provide significant performance improvements over existing prime number generation methods. Our proposed algorithms not only surpass traditional algorithms in terms of execution time but also demonstrate the unique capability to generate prime numbers from range to range with a commendable performance. Moreover, we explore our algorithms’ mathematical foundations and underlying principles, providing a clear understanding of their inner workings and the basis for their performance improvements. Our in-depth analysis allows us to showcase the benefits and advantages of our proposed algorithms over existing methods, demonstrating their potential impact on a wide array of applications. The contribution of the paper is indicated as follows:

1. The paper introduces two novel algorithms for generating prime numbers that are based on the formula of odd-composed numbers.
2. The proposed algorithms exhibit remarkable performance improvements in generating prime numbers compared to existing algorithms, particularly for generating prime numbers within a specified range and large maximum prime numbers.
3. The algorithms are more efficient in generating prime numbers within moderate ranges and have a unique capability to find prime numbers within a given range, setting them apart from traditional methods.
4. The performance enhancement and adaptability of these algorithms can significantly impact various applications that depend on prime numbers, including cryptographic systems and distributed computing.
5. The algorithms offer an efficient and flexible method for generating prime numbers, which can facilitate the development of more secure and reliable communication systems, enable faster computations in number theory, and support advanced computer science and mathematics research.

The remainder of this paper is structured as follows: Section 2 provides a comprehensive background on prime numbers, their properties, and existing prime number generation algorithms, as well as their respective strengths and weaknesses. Section 3 introduces the formulas of odd-composed numbers and details the development of our proposed algorithms, along with a step-by-step explanation of their implementation. Section 4 presents the experimental results that demonstrate the performance of our algorithms in comparison to other prime number generation methods, highlighting the improvements in execution time and range-based generation capabilities. Finally, Section 5 concludes the paper and discusses the potential impact of our algorithms on various fields and applications, as well as future research directions and enhancements to improve their performance further.

## 2. Background and related work

The quality of prime number generation can be measured using different methods based on the complexity of time and space, the accuracy of generating composite numbers, the output regularity, and the number of bits required to produce the prime numbers [5]. In this section, we explained prime numbers, their properties and applications, and the most used algorithms for generating prime numbers.

### 2.1. Prime numbers and their properties

A prime number is a natural number greater than 1 that has no divisors other than 1 and itself. The first few prime numbers are 2, 3, 5, 7, 11, 13, 17, and so on [6]. The generation of a prime factor from the prime number is based on dividing the prime number by another number, where each prime number has a unique set of prime factors. Prime numbers have several unique properties that make them the subject of intense study in number theory and other branches of mathematics. Some of the most important properties of prime numbers are:

- Every natural number greater than 1 can be factored into a unique product of prime numbers. This is known as the Fundamental Theorem of Arithmetic.
- There are an infinite number of prime numbers. This was first proved by Euclid more than 2,000 years ago.
- The distribution of prime numbers becomes less dense as numbers get larger. The Prime Number Theorem states that the number of primes less than or equal to a given number ’*x*’ is approximately equal to *x*/*ln*(*x*) where *ln*(*x*) is the natural algorithm of ’*x*’.
- Some conjectures about prime numbers, such as the Goldbach Conjecture and the Twin Prime Conjecture, remain unproven despite extensive computational evidence supporting their validity.

### 2.2. Prime number applications

The applications of prime numbers are classified into various topics based on the objectives. Cryptography applications are considered the most widely used topics that apply prime numbers [5–10]. The prime numbers are used for creating secure encryption keys where it is difficult to factor the generated large numbers into their prime factors. Public key cryptography uses public and private keys to encrypt the message between the sender and receiver. It is difficult to factor large numbers into their prime factors, and therefore, the difficulty of predicting the private key from intruders will increase. The prime numbers can generate unpredictable numbers embedded into different algorithms to produce one-time-password (OTP) applied in recent security applications.

The prime numbers can also be generated using genetic algorithms (GA) [11], where the population’s chromosomes represent the prime numbers. The GA’s fitness function calculates the likelihood that a certain chromosome has a prime number. Only the fittest chromosomes are chosen to procreate. After being evaluated, the best progeny of the chosen chromosomes is then chosen for reproduction. This procedure is repeated until a prime number is discovered.

A prime number is a powerful tool for securing communication [12], such as Diffie-Hellman key exchange and the Elliptic curve that create secure communication channels between the two parties. The prime number can also be used in digital signatures for authenticating messages between the sender and receiver. The prime numbers can also produce pseudorandom numbers that can be applied in number theory, gaming, and simulations [13]. In number theory, the characteristics of other mathematical entities, such as polynomials and matrices, are also studied using prime numbers [14,15]. For instance, it is possible to demonstrate using the Fundamental Theorem of Arithmetic that every polynomial with integer coefficients may be factored into a sum of prime polynomials.

### 2.3. Existing prime number generation algorithms

A certain prime number generation algorithm is chosen depending on the particular application. For instance, the sieve of Eratosthenes is a good option if you need to produce many prime numbers. The sieve of Sundaram or the sieve of Atkin would be preferable if you need to generate a few prime numbers. Over the years, numerous algorithms have been developed for generating prime numbers. Some of the most well-known algorithms are:

#### 2.3.1 Sieve of Eratosthenes

This method is a traditional algorithm for finding all prime numbers up to a given limit. It works by iteratively marking the multiples of each prime number starting from 2, and the remaining unmarked numbers are prime numbers. The algorithm has a time complexity of *O*(*n log*(*log n*)) and space complexity of *O*(*n*), making it one of the most efficient algorithms for generating prime numbers up to a given limit [16].

However, it could be better-suited for generating prime numbers within a specific range or for parallel execution on modern multi-core processors [17]. For example, if it is required to find the prime numbers between 150 and 250, the Sieve of Eratosthenes method will start finding the primes from 1 to 250, although the required limit is 150 to 250. As a result, it is not cache friendly method as it consumes more time to generate the required primes.

#### 2.3.2. Sieve of Sundaram

The Sieve of Sundaram is another sieve-based algorithm for finding prime numbers up to a given limit. The objective of this method is based on identifying the odd numbers from 1 to a specific limit and then removing all the numbers that are the sum of two successive primes. Therefore, the remaining numbers will be the prime numbers. Different formulas are applied to explore the method to generate the primes based on a unified framework [18]. The method eliminates numbers of the form *x*+*y*+2*xy* where *x* and *y* are integers such that 1≤*x*≤*y*. The remaining numbers in the list, when doubled and incremented by 1, are prime numbers. The Sieve of Sundaram has a time complexity of *O*(*n* log *n*) and space complexity of *O*(*n*), making it less efficient than the Sieve of Eratosthenes for generating prime numbers up to a given limit.

#### 2.3.3. Sieve of Atkin

The Sieve of Atkin is a modern sieve-based algorithm for finding prime numbers up to a given limit [19,20]. It is an optimized version of the Sieve of Eratosthenes, with improved time complexity of *O*(*n log* (*log n*)) and space complexity of$O(n^{\frac{1}{2}}\times \log\;n)$. The Sieve of Atkin is more efficient than the Sieve of Eratosthenes and the Sieve of Sundaram for generating prime numbers up to a given limit but could be better-suited for generating prime numbers within a specific range. This method is faster than previous methods and can generate primes with a wider range.

#### 2.3.4. Miller-Rabin primality test

The Miller-Rabin primality test is a probabilistic algorithm for determining whether a given number is prime or composite [20]. The for checking whether the number is prime or composite, the Miller-Rabin checks a random number *x* in the range from 2 to *m*−2 and then raise *x* to the power *m*−1 based on the formula *x*^*m*−1^ *mod m*. If the result is 1 then the number *m* is prime; otherwise the number *m* is composite. Therefore, the method is based on the observation that if a number is prime, certain properties hold true for its modular arithmetic. The algorithm has a time complexity of *O*(*k* log *n*), where *k* is the number of iterations, and is particularly useful for testing the primality of large numbers. However, it is not designed to generate prime numbers directly, and must be combined with other techniques to identify primes.

#### 2.3.5. AKS primality test

The AKS primality test is a deterministic algorithm for checking whether a given number is prime or not. The main mechanism is based on using the property of a polynomial to verify if the number is composite and must have a factor that is smaller than its square root [21]. The AKS algorithm checks all numbers that are smaller than the square root of a specified number. If the resulted numbers contain no factors, then the specified number is prime and vice versa. The time complexity of AKS is$O(log\;n^{\frac{21}{2}})$. Other research methodologies [22] stated that the time complexity of AKS is as low as *O*(*log n*^6^). Although the AKS is slower than the Miller-Rabin primality test, it provides a definitive answer to whether a number is prime or composite. Like the Miller-Rabin test, the AKS test is not designed for prime number generation and must be combined with other techniques for this purpose. As presented in Table 1, a comparison of different prime number methods with their objectives, complexity, and limitations is provided.

**Table 1:** Prime number methods and their time complexity.

Ref	Method	Objective	Complexity	Limitation
[17]	Sieve of Eratosthenes	Generating prime numbers up to a specified limit using a straightforward and effective approach.	Time Complexity: *O*(*n log*(*log n*))	Specified to certain limit. High memory usage.
[18]	Sieve of Sundaram	Generating prime numbers with a small limit.	Time Complexity: *O*(*n* log *n*)	Specified to certain limit. Cannot identify primes that are not part of odd numbers.
[20]	Sieve of Atkin	It is a powerful tool for generating primes with a wide range.	Time Complexity: *O*(*n log*(*log n*))	More complex. High memory usage.
[23]	Miller-Rabin	A probabilistic method for determining whether a given number is prime or composite.	Time Complexity: *O*(*k* log *n*)	Probabilistic method that can produce false positive numbers.
[21]	AKS	A deterministic method to verify whether a specified number is prime or composite.	Time Complexity: *O*(*log n*^6^)	Difficult to implement. More complex.

### 2.4 Prime number generation in specific ranges

Generating prime numbers within a specific range is more challenging than generating prime numbers up to a given limit. While sieve-based algorithms such as the Sieve of Eratosthenes, the Sieve of Sundaram, and the Sieve of Atkin are highly efficient for generating prime numbers up to a given limit, they are not well-suited for generating prime numbers within a specific range, as they require significant modifications to adapt to this problem. Several approaches have been proposed for generating prime numbers within specific ranges. Some of these approaches involve primality tests such as the Miller-Rabin test or the AKS test combined with other techniques for generating potential prime candidates. However, these methods are less efficient than sieve-based algorithms for generating prime numbers up to a given limit. The choice of a specific range method depends on the application’s requirements. For example, the Sieve of Eratosthenes is a very efficient algorithm for generating prime numbers in a large range, but it can be slow for generating prime numbers in a small range. The Miller-Rabin Primality Test is a more accurate algorithm than the Sieve of Eratosthenes but also slower. The AKS Primality Test is the most accurate algorithm but also the slowest.

## 3. Prime number theory and cryptography

Prime numbers play a crucial role in cryptography, particularly in encryption algorithms like RSA and Diffie-Helman key exchange for key management. Different enhanced models are proposed to secure applications especially with different authentication factors [24] and the use of enhanced prime-based models becomes crucial due to the increase of security breaches and brute-force attacks. This section would delve into the fundamental principles of prime numbers and their significance in cryptographic systems. Prime numbers, due to their unique properties, form the backbone of many cryptographic algorithms.

In RSA, one of the most widely used public-key cryptosystems, the security relies on the computational difficulty of factoring the product of two large prime numbers. The RSA algorithm begins with the selection of two large prime numbers, which are kept secret. These primes are used to generate a public key and a private key. The public key can be widely distributed, while the private key is kept secret. The security of RSA hinges on the principle that while it’s relatively easy to multiply two large primes, it’s exceedingly difficult to factor their product back into the original primes. This asymmetry is what makes RSA secure. The larger the primes, the more secure the encryption, as the difficulty of factoring increases exponentially with the size of the primes. Moreover, the randomness and unpredictability of prime numbers are vital. Primes used in cryptography must be generated randomly and be of a sufficient size to prevent predictable patterns, which could be exploited by attackers. With the advent of quantum computing, traditional cryptographic systems that rely on prime numbers could be vulnerable, highlighting the need for ongoing research and development in cryptographic methods that can withstand future technological advances. Behind the hardness of factoring, prime numbers have several mathematical properties, such as randomness and quadratic residues, which make RSA strong. Cryptography is however vulnerable to side-channel attacks and it seems no hope for post-quantum cryptography (PQC). Blinding and PQC research however presents hope. Secure key management practices and investigating prime numbers similar to Einstein primes are constrintential for preempting. Identifying the role of society in privacy and the ethical implications, such as key escrow, is critical in the ever-changing cyber landscape.

The Diffie-Hellman protocol employs large prime numbers and a primitive root modulo a prime. Each party than picks up a private key, which must remain secret, and hence the public key used in the verification process is generated by using the prime chosen in modular arithmetic with the primitive root. Next, the public keys are exchanged; then each party computes the shared secret key from the received public key and the private key. This protocol uses discrete logarithm problem as mathematical basis, which makes it computationally infeasible to find out the secret key even when public keys are intercepted. The Diffie-Hellman key exchange security is similar to RSA and is dependent on prime number properties. Employment of large primes is the feature of the public channel which makes the communication secure, because difficulty of the discrete logarithm problem grows together with the size of the prime modulus. Unpredictability and randomness of prime numbers are the basic requirements which work to avoid deliberate attacks, for example those using especially created patterns during the key generations.

Diffie-Hellman, once considered secure against traditional computers, now faces serious threats from rapidly advancing processing power and the dawn of quantum computing. Its security might crumble in the face of these advancements. We must diligently analyze and refine cryptographic methods like Diffie-Hellman and RSA, ensuring they remain robust against the ever-evolving technological landscape.

While the primary focus of our research has been on the use of prime number generation algorithms in RSA encryption, these algorithms have several other applications across various domains. Here, we discuss potential scenarios where our algorithms could be beneficial.

### 3.1. Cryptographic protocols

Apart from RSA encryption, prime numbers are fundamental to various other cryptographic protocols:

- Diffie-Hellman Key Exchange:

Prime numbers are used to establish a shared secret between two parties over an insecure channel. The efficiency of prime number generation can enhance the security and speed of key exchanges.

- ElGamal Encryption:

This public-key cryptosystem also relies on large prime numbers for its security. Efficient prime generation improves the overall performance of the encryption and decryption processes.

### 3.2 Secure communication systems

Prime numbers play a crucial role in securing communication systems:

- Digital Signatures:

Algorithms like DSA (Digital Signature Algorithm) require prime numbers for generating secure digital signatures. Fast prime number generation can enhance the efficiency of signing and verifying digital documents.

- Pseudorandom Number Generators (PRNGs):

Prime numbers are used in PRNGs to generate secure and unpredictable sequences of numbers. Applications in secure communication, gaming, and simulations benefit from efficient prime generation.

### 3.3 Computational number theory

Prime number generation is essential in various number-theoretic problems and mathematical research:

- Prime Testing and Factorization:

Efficient algorithms for generating primes assist in testing the primality of numbers and in factorizing large numbers, which are central problems in computational number theory.

- Mathematical Conjectures:

Research in prime numbers supports the study and verification of conjectures such as the Goldbach Conjecture and the Twin Prime Conjecture. Generating large primes helps in providing computational evidence for these conjectures.

### 3.4. Security applications

Prime numbers enhance security in various applications beyond traditional cryptographic systems:

- Secure Multiparty Computation:

In protocols where multiple parties compute a function securely, prime numbers ensure that intermediate computations remain secure and private.

- Zero-Knowledge Proofs:

These cryptographic protocols, which allow one party to prove to another that a statement is true without revealing any information, often use prime numbers to maintain security and privacy.

Our proposed algorithms use secure random number generators (RNGs) to seed the prime generation process. These RNGs are designed to provide cryptographically secure seeds, ensuring the unpredictability of the generated primes. The proposed algorithms have been carefully designed and evaluated to ensure their security and robustness, particularly in cryptographic applications. By incorporating secure random number generation, rigorous primality testing, and secure implementation practices, we aim to mitigate potential vulnerabilities and provide reliable prime generation for cryptographic systems.

In a final analysis, our proposed algorithms for prime number generation contributes to futureproofing cryptography against threats like quantum computing, but remember, robust key management and exploring alternative solutions like Post-Quantum Cryptography are equally crucial. Ultimately, your work is a significant step in the ongoing effort to secure our digital world.

## 4. Proposed algorithms

This paper proposes a novel prime number generation algorithm that efficiently generates prime numbers within a specific range. The algorithm is based on relationships between odd composite numbers and their factors and outperforms other prime generation algorithms in speed and performance. We present two algorithms, Algorithm 1 (Prime Number Generation) and Algorithm 2 (Prime Number Generation within a Range), that implement the proposed method.

### 4.1. Algorithm 1: Prime number generation within a limit

As presented in Algorithm 1, the prime number generation algorithm takes a single parameter called a limit *L*, and is responsible for generating prime numbers up to the given limit. The following formula generates the odd composite number as follows:

$$m=\sum_{n=1}^{N}4n^{2}+4n+1+\sum_{l=0}^{N_{1}}l(4n+2)$$ (1)

The algorithm starts by calculating the values of *N* and *N*_1_ then *N* and *N*_1_ can be determined using the following formulas:

$$N=round(\sqrt{2\;y}+1)$$ (2)

$$N_{1}=round\left(\frac{2y-9}{6}\right)$$ (3)

Given that *L* = 2*y* is the last number in interval and *m*<*L*

**Table:** 

**Algorithm 1. Prime Number Generation within a Limit.**	
**Input** → Limit	
**Output** ← List of prime numbers up to limit *L*	
1	*y* = L/*2*
2	$N=\sqrt{\left(2\times y\right)}+1$
3	*N*_1_ = ((2×*y*)−9)/6
4	Initialize list b with all odd numbers in the range [1,L]
5	Initialize an empty list prime_list
6	*For n in range* [1,*N*]
7	$m=\sum_{n=1}^{N}4n^{2}+4n+1+\sum_{l=0}^{N_{1}}l(4n+2)$
8	*add m to prime*_*list*_
9	*prime*_*numbers*_ = *b*−*empty*_*list*_
10	*Return prime*_*numbers*_

The algorithm initializes a list b with all odd numbers in the range [1, L]. It then iterates through each value of *n* in the range [1, *N*], and for each *n*, it calculates the odd composite number *m* based on Formula (1). To explain Algorithm 1 in detail, the following example summarizes the overall process for generating the prime numbers based on a limit.

**Example 1**: **(Prime Generation within a limit)**: if we want to find the prime numbers from [1,100].

**Step 1:** Calculate the set of odd numbers in the range [1,100] using the Formula 2 *n*+1 and then removes 1 from the generated set as explained in Table 2.

**Table 2:** Generation of odd numbers.

1	3	5	7	9
11	13	15	17	19
21	23	25	27	29
31	33	35	37	39
41	43	45	47	49
51	53	55	57	59
61	63	65	67	69
71	73	75	77	79
81	83	85	87	89
91	93	95	97	99

**Step 2:** Given that *y* = 50 then

$$N=round(\sqrt{100}+1)=11$$

$$N_{1}=round(\frac{100-9}{6})=15$$

**Step 3:** The generation of odd composite numbers are calculated based on Formula (1) as follows:

$$m=\sum_{n=1}^{11}4n^{2}+4n+1+\sum_{l=0}^{16}l(4n+2)$$

The resulting numbers from the formula are removed to generate the prime numbers in Table 3.

**Table 3:** Generation of prime numbers based on a limit.

1	3	5	7	9
11	13	15	17	19
21	23	25	27	29
31	33	35	37	39
41	43	45	47	49
51	53	55	57	59
61	63	65	67	69
71	73	75	77	79
81	83	85	87	89
91	93	95	97	99

The list of odd composite numbers, `prim_list`, is created by adding all `m`values obtained in this process. Finally, the algorithm returns the prime numbers by eliminate the set of odd composite numbers from the set of odd numbers in the range, and list the result set with number 2 as the first number.

- Given that *n* = 1 and *l* = 0 then *m* = 9. The value of *N*_1_ refers to the multiply of 3 by odd number. The removed numbers are [9–15–21–27–33–39–45–51–57–63–69–75–81–87–93–99].
- Given that *n* = 2 and *l* = 0 then *m* = 25. The value of *N*_1_ refers to the multiply of 5 by odd number. The removed numbers are [25–35–55–65–85–95].
- When *l* = 8 then *m* = 105, we stop calculated because m must be less than 2y (or *m*<2*y*)
- Given that *n* = 3 and *l* = 0 then *m* = 49. The value of *N*_1_ refers to the multiply of 7 by odd number. The removed numbers are [49–77–91].

When *l* = 4 then *m* = 105, we stop calculated because m must be less than 2y (or *m*<2*y*).

Finally, the result set of prime number with the number 2 is:

[2,3,5,7,11,13,17,19,23,29,31,37,41,43,47,53,59,61,67,71,73,79,83,89,97].

### 4.2. Algorithm 2: Prime number generation within a range

In cryptography, generating prime numbers inside a given range is essential, especially for techniques like RSA. The efficiency of key pair generation is increased by limiting the generation to a certain range, and the security of RSA is predicated on the difficulty of factoring the product of two large prime numbers. The algorithm’s cryptographic strength is guaranteed by choosing primes that fall inside this range. Moreover, defining a range improves the unpredictability and security of cryptographic keys and initialization vectors in cryptographic protocols that use random prime number generation. One of the most important steps in strengthening the cryptographic foundations of different security protocols is the careful examination of a specific range in prime number creation.

As proposed in Algorithm 2, the prime number generation within a range algorithm takes two parameters, `start`and `end`, and generates prime numbers within the specified range or interval [*a*,*b*] where *a* is the start of the interval and *b* is the end of the interval as presented in Formulas (4) and (5).

**Table:** 

**Algorithm 2. Prime Number Generation within a Range.**	
**Input** → Start (*a*), End (*b*)	
**Output** ← List of prime numbers within the specified range [a, b]	
1	*y* = *a*/2
2	*y*_1_ = *b*/2
3	$x_{1}=round(\sqrt{2\times y})$
4	$N=round(\sqrt{2\times y_{1}})$
5	*N*_1_((2×*y*_1_)−9)/6
6	*A*_1_ = *empty*_*list*_
7	*B*_1_ = *empty*_*list*_
8	*for n in range* []
9	$m=\sum_{n=x_{1}}^{N}4n^{2}+4n+1+\sum_{l=0}^{N_{1}}l(4n+2)$
10	*$m_{1}=\sum_{n_{1}=1}^{N}4{n_{1}}^{2}+4n_{1}+1+\sum_{l_{1}=g}^{N_{1}}l_{1}(4n_{1}+2)$*
11	$g=\sum_{n_{1}=1}^{N}\left|round(\frac{y}{2n_{1}+1}-n_{1})\right|$
12	*add m to A*_1_
13	*add m*_*1*_ *to B*_1_
14	*prime*_*numbers*_ = *B*_1_−*A*_1_
15	*Return prime*_*numbers*_ *within a Range*

The algorithm initializes two empty lists, `A1`and `B1`, to store the odd composite numbers in the specified range. The algorithm iterates through each value of `*n*`in the range `[0,*N*] `, and for each `*n*`, it calculates the odd composite numbers `*m* `and `*m*_1_`using the following formulas:

$$m=\sum_{n=x_{1}}^{N}4n^{2}+4n+1+\sum_{l=0}^{N_{1}}l\left(4n+2\right)$$ (4)

$$m_{1}=\sum_{n_{1}=1}^{N}4{n_{1}}^{2}+4n_{1}+1+\sum_{l_{1}=g}^{N_{1}}l_{1}(4n_{1}+2)$$ (5)

$$y=\frac{a}{2}$$ (6)

$$y_{1}=\frac{b}{2}$$ (7)

Where *a* is the start of the range and *b* is the end of the range. The remaining parameters in the formulas can be determined as follows:

$$x_{1}=round\left(\frac{\sqrt{2y}}{2}\right)$$ (8)

$$N=round(\sqrt{2y_{1}})$$ (9)

$$N_{1}=round(\frac{2y_{1}-9}{6})$$ (10)

$$g=\sum_{n_{1}=1}^{N}\left|round(\frac{y}{2n_{1}+1}-n_{1})\right|$$ (11)

The code will be stop when *m*_1_>2*y*

To explain Algorithm 2 in detail, the following example summarizes the overall process for generating the prime numbers based on a range.

**Example 2**: **(Prime Generation within a range)**: if we want to find the prime numbers from [200,400].

**Step 1:** identify the start value of the range *a* = 200 and end value of the range *b* = 400

**Step 2:** identify the value of *y* = 100 and *y*_1_ = 200

**Step 3:** calculate the parameters *x*_1_,*N*_1_,*g* as follows:

$$x_{1}=round(\frac{\sqrt{200}}{2})=7$$

$$N=round(\sqrt{400})=20$$

$$N_{1}=round(\frac{2*200-9}{6})=65$$

$$g=\sum_{n_{1}=1}^{20}\left|round(\frac{100}{2n_{1}+1}-n_{1})\right|$$

**Step 4:** calculate the odd composite numbers *m* as follows:

$$m=\sum_{n=7}^{20}4n^{2}+4n+1+\sum_{l=0}^{65}l(4n+2)$$ (12)

Due to the equality of = *x*_1_, the value of *n* = 7, the following parameters will be extracted to find the odd composite numbers.

Given that *n* = 7 and *l* = 0 then m = 225. The value of *N*_1_ refers to the multiply of 15 by odd number. The removed numbers are [225−255−285−315−345−375].

Given that *n* = 8 and *l* = 0 then *m* = 289. The value of *N*_1_ refers to the multiply of 17 by odd number. The removed numbers are [289−323−357−391].

Then store all odd composite numbers generated from Eq (12) in the set A1.

**Step 5:** identify the value of *g* based on Formula (11) as follows:

$$g=\sum_{n_{1}=1}^{20}\left|round(\frac{100}{2n_{1}+1}-n_{1})\right|$$

The algorithm then eliminates the set of odd composite numbers obtained from `*m*_1_`from the set of odd composite numbers obtained from `*m*`to get the prime numbers within the specified range as follows:

$$m_{1}=\sum_{n_{1}=1}^{20}4{n_{1}}^{2}+4n_{1}+1+\sum_{l1=g}^{65}l_{1}(4n_{1}+2)$$

*n*_1_ = 1,*g* = 32,*l*_1_ = 32: 66 multiply of 3 by odd number from 69 to 133.

- Given that $n_{1}=1,\;g=32,\;and\;l_{1}=32:66$. The value of *N*_1_ refers to the multiply of 3 by odd number from 69 to 133. The removed numbers are [].
- Given that $n_{1}=2,\;g=18,\;and\;l_{1}=18:38$. The value of *N*_1_ refers to the multiply of 5 by odd number from 41 to 79. The removed numbers are [].
- Given that $n_{1}=3,\;g=11,\;and\;l_{1}=11:26$. The value of *N*_1_ refers to the multiply of 7 by odd number from 29 to 57. The removed numbers are [203−217−231…399].

Then store all odd composite numbers generated from Eq (13) in the set B1.

We combine the two sets A1 and B1 in the set A which represent all odd composite numbers in the specified range.

Finally, the algorithm returns the prime numbers by eliminate the set A of odd composite numbers from the set of odd numbers in the range. The resulting values are presented in Table 4.

**Table 4:** Generation of prime numbers based on a range.

201	203	205	207	209
211	213	215	217	219
221	223	225	227	229
231	233	235	237	239
241	243	245	247	249
251	253	255	257	259
261	263	265	267	269
271	273	275	277	279
281	283	285	287	289
291	293	295	297	299
301	303	305	307	309
311	313	315	317	319
321	323	325	327	329
331	333	335	337	339
341	343	345	347	349
351	353	355	357	359
361	363	365	367	369
371	373	375	377	379
381	383	385	387	389
391	393	395	397	399

### 4.3. Time and space complexity analysis

The time complexity of the proposed algorithms can be analyzed based on the operations performed. Based on the prime generation within a limit presented in Algorithm 1, the main loop iterates for *N* times, where *N* is approximately equal to$\sqrt{\left(2\times y\right)}$. The time complexity of Algorithm 1 can be analyzed as follows:

1. Initializing the list of odd numbers in the range [1,*L*] takes *O*(*L*/2) time.
2. The outer loop runs *N* times, where *N*≈2*y*+1
3. Inside the loop, generating each odd composite number *m*m involves constant-time operations.

Therefore, the overall time complexity for Algorithm 1 is approximatelyO(N)=O(2y+1)=O(L).

Based on the prime generation within a range presented in Algorithm 2, the main loop iterates for *x* times, where *x* is approximately equal to$\sqrt{\left(2\times y_{1}\right)}$. Similar to Algorithm 1, there is an inner loop that iterates for *N*_1_ times, where *N*_1_ is approximately equal to ((2×*y*_1_)−9)/6. The time complexity of Algorithm 2 can be analyzed as follows:

1. Initializing the empty lists *A*1 and *B*1 involves constant-time operations.
2. The outer loop runs *N* times, where *N*≈2*y*_1_
3. Inside the loop, generating each odd composite number *m* and *m*1 involves constant-time operations.

Therefore, the overall time complexity for Algorithm 2 is approximately *O*(*N*) = *O*(2*y*_1_) = *O*(*b*)

It is essential to note that the time complexity of the proposed algorithms mainly depends on the size of the range (`y`for Algorithm 1 and `*y*_1_ `for Algorithm 2). The algorithms perform well, especially when generating prime numbers within a moderate range, with acceptable performance. The space complexity of Algorithm 1 can be analyzed as follows:

1. The list of odd numbers in the range [1, L] requires *O*(*L*/2) space.
2. The list of prime numbers (prime_list) also requires *O*(*L*/2) space.

Therefore, the overall space complexity for Algorithm 1 is *O*(*L*).

The space complexity of Algorithm 2 can be analyzed as follows:

1. The lists A1 and B1 store odd composite numbers, requiring O (b−a) space.

Therefore, the overall space complexity for Algorithm 2 is *O* (*b*−*a*)

### 4.4. Algorithms comparison

The proposed algorithms, Algorithms 1 and 2, have distinct advantages compared to other prime number generation algorithms, such as the Sieve of Eratosthenes and the Sieve of Sundaram. One of the primary benefits is their capability to generate prime numbers within a specified range, which is particularly useful for applications requiring prime numbers from a certain interval. Furthermore, the proposed algorithms demonstrate acceptable performance, especially when generating prime numbers within a moderate range. This is due to the relationship between odd composite numbers and the factors the algorithms exploit to identify prime numbers efficiently.

Algorithm 1 and Algorithm 2 are both effective algorithms for generating prime numbers. They have several advantages over other algorithms, such as generating prime numbers within a specified range and demonstrating acceptable performance when generating prime numbers within a moderate range. The authors of [25] proposed a new algorithm for generating prime numbers that use a probabilistic primality test. The algorithm is considered efficient for generating prime numbers within a specified range. The algorithm is not deterministic, so there is a small chance it will incorrectly identify a composite number as prime.

As presented in [26], a new prime number generator algorithm is generated based on the wheel sieve. The generator is faster than the Sieve of Eratosthenes and the Sieve of Sundaram for generating prime numbers within a moderate range. In this paper, the generator is very fast for generating prime numbers within a moderate range. However, the generator could more efficiently generate prime numbers within a large range. The authors of [27] proposed a new prime number generator based on the Sieve of Atkin. The generator is efficient for generating prime numbers within a large range. The generator is very efficient for generating prime numbers within a large range, but it is more complex to implement than the other two. In order to explore the effectiveness of the algorithm 1 and 2, Table 5 explains a comparison between recent algorithms with the proposed algorithms.

**Table 5:** Comparative analysis of proposed algorithms with state-of-the-art prime algorithms.

Algorithm	Time Complexity	Space Complexity	Range-Based Generation	Performance in Cryptographic Applications
Sieve of Eratosthenes	*O*(*n log log n*)	*O*(*n*)	Not well-suited	Moderate
Sieve of Sundaram	*O*(*n log n*)	*O*(*n*)	Not well-suited	Moderate
Sieve of Atkin	*O*(*n log log n*)	*O*(*n*^1/2^*log n*)	Not well-suited	Moderate
Miller-Rabin Primality	*O*(*k log n*)	*O*(1)	Can be adapted	High (probabilistic)
AKS Primality Test	*O*(*log*^6^ *n*)	*O*(*log*^3^ *n*)	Not well-suited	High (deterministic)
**Proposed Algorithm 1**	*O*(*L*)	*O*(*L*)	Well-suited	High
**Proposed Algorithm 2**	*O*(*b*)	*O*(*b*−*a*)	Well-suited	High

### 4.5. Handling edge cases and boundary conditions

In this section, the proposed algorithms are applied to handle edge cases and boundary conditions to verify the robustness and reliability of algorithms as follows:

#### Edge cases and boundary conditions for Algorithm 1

- Small Limits (e.g., L < 10)
- For very small limits, the algorithm ensures that all prime numbers within the given limit are correctly identified. The algorithm initializes and processes small ranges efficiently.
- Limit Equals 1 or 2
- When *L* = 1, the algorithm returns an empty list since there are no prime numbers less than {2}.
- When *L* = 2, the algorithm correctly returns the single prime number with a value of {2}.
- Non-Prime Limits
- The algorithm handles non-prime limits by correctly identifying and excluding composite numbers. For example, if *L* = 10, the algorithm returns {2, 3, 5, 7}, excluding the non-prime {10}.
- Robustness and Reliability
- The algorithm’s design ensures robustness by handling all edge cases gracefully. It correctly initializes, processes, and filters primes within the given limit, maintaining accuracy even for small or boundary input values.

#### 4.5.2 Edge cases and boundary conditions for Algorithm 2

- Empty Range (e.g., a > b)
- If the start of the range *a* is greater than the end of the range *b*, the algorithm returns an empty list, indicating no primes in an invalid range.
- Single Number Range (e.g., a = b)
- When *a* = *b*, the algorithm checks if the single number is prime and returns it. For example, if *a* = *b* =17, the algorithm returns the value of {17}.
- Range with No Primes
- The algorithm correctly identifies ranges that contain no prime numbers. For example, for the range {14, 16}, it returns an empty list since there are no primes between 14 and 16.
- Large Ranges
- The algorithm efficiently handles large ranges by maintaining its linear time complexity, ensuring it can scale to larger inputs without performance degradation.
- Robustness and Reliability
- The algorithm’s structure ensures robustness by validating the input range and handling special cases appropriately. It maintains reliability by accurately generating prime numbers within valid ranges, even when the range contains only one number or no primes at all.

### 4.6 Error handling

Our proposed algorithms include several mechanisms to handle potential errors and exceptional cases effectively as follows:

- Input Validation:
- Range and Limit Checks: Before processing, the algorithms validate the input range or limit to ensure they are within acceptable bounds. If the input is invalid (e.g., negative values, non-integer inputs), the algorithms return an appropriate error message or handle the input gracefully.
- Boundary Conditions: The algorithms handle boundary conditions, such as the smallest possible limit (1) and ranges where the start value is greater than the end value, by returning an empty list of primes or adjusting the range appropriately.
- Primality Testing Errors:
- False Positives: In probabilistic primality tests, there is a small chance of false positives. To mitigate this, our algorithms use multiple rounds of testing to reduce the probability of error. For deterministic tests, additional checks are implemented to ensure the accuracy of the results.
- Overflow and Underflow: For very large numbers, there is a risk of overflow or underflow errors. Our algorithms include safeguards to handle such cases by using appropriate data types and arithmetic operations that support large integers.
- Resource Management:
- Memory Allocation Errors: The algorithms monitor memory usage and include error handling for cases where memory allocation fails. This ensures that the algorithms do not crash due to insufficient memory.
- Timeouts: For extremely large inputs, the algorithms include timeout mechanisms to prevent excessive computation time. If an operation exceeds the predefined time limit, the algorithm terminates gracefully and returns a partial result or an error message.
- Algorithm-Specific Error Handling:
- Algorithm 1 (Limit-Based Generation): Handles cases where the limit is very small or very large by adjusting the internal parameters and ensuring that the prime generation process completes successfully.
- Algorithm 2 (Range-Based Generation): Manages cases where the range is invalid or spans a very large interval by segmenting the range into smaller, manageable parts and processing each part sequentially.

### 4.7 Robustness and resilience

Our proposed algorithms are designed to be robust and resilient to input variations and unexpected conditions. The following features contribute to their robustness:

- Dynamic Adaptation:
- The algorithms dynamically adapt their internal parameters based on the input size and characteristics. This ensures that they maintain optimal performance and accuracy regardless of the specific input values.
- Error Logging and Reporting:
- Comprehensive error logging and reporting mechanisms are integrated into the algorithms. This allows for detailed tracking of errors and facilitates debugging and improvement of the algorithms.
- Graceful Degradation:
- In cases where errors or unexpected conditions occur, the algorithms degrade gracefully. Instead of failing abruptly, they return partial results or informative error messages, ensuring that users receive meaningful output even in adverse conditions.
- Extensive Testing:
- The algorithms have been extensively tested under various scenarios, including edge cases and stress conditions. This rigorous testing ensures that they can handle a wide range of inputs and maintain their reliability and performance.

### 4.8. Performance optimization

To further optimize the performance of the proposed algorithms, several techniques can be employed:

1. **Parallelization:** The calculations for `*m*`and `*m*_1_`in Algorithm 2 can be executed concurrently, which can significantly speed up the algorithm’s execution when processing large input ranges.
2. **Memory optimization:** The algorithms can be optimized to use less memory by employing data structures such as bit arrays to represent the odd composite numbers and prime numbers.
3. **Incremental computation:** The algorithms can be modified to compute the prime numbers incrementally instead of generating them all at once. This approach is useful when the required prime numbers are needed one at a time or when the complete list of prime numbers is not necessary.

Algorithmic optimizations can significantly enhance the efficiency of algorithms through various techniques. Parallelization can be implemented via data parallelism, where the input range is divided into smaller, concurrently processed segments, and task parallelism, which separates algorithm tasks into parallel processes to optimize CPU usage. Incremental sieve updates, such as dynamic sieve adjustment, incrementally update the sieve as new ranges are processed, saving computational resources and memory. Memory-efficient data structures, like bitwise operations and sparse data structures (e.g., hash sets, bloom filters), can reduce memory usage. Mathematical optimizations include advanced prime-checking techniques like the Baillie-PSW test and using the Number Theoretic Transform (NTT) for fast polynomial multiplication. Analyzing computational complexity reveals that segmented sieves and wheel factorization can reduce time complexity, while compressed data structures and lazy evaluation can lower memory usage by only generating primes as needed.

In conclusion, the proposed algorithms offer a new and efficient approach to prime number generation within a specified range. The algorithms leverage relationships between odd composite numbers and their factors to identify prime numbers effectively, resulting in acceptable performance and outperforming other prime generation algorithms in specific scenarios.

## 5. Mathematical models for prime number algorithms in cryptography

In this paper, two proposed algorithms are implemented for generating prime numbers: Algorithm 1 (Prime Number Generation within a Limit) and Algorithm 2 (Prime Number Generation within a Range). Algorithm 1 takes a single parameter, limit, which is the maximum number to be considered. It generates all prime numbers up to limit. Algorithm 2 takes two parameters, start and end, which specify the range of numbers to be considered. It generates all prime numbers within the specified range.

Both algorithms work by first generating all odd composite numbers in the specified range. An odd composite number is a number that is odd and not prime. Once all odd composite numbers have been generated, the algorithms use a relationship between odd composite numbers and their factors to identify the prime numbers in the range. This relationship states that the difference between two odd composite numbers is always even and divisible by 2. The algorithms use this relationship to quickly identify the prime numbers in the range, which significantly improves their speed and performance. To incorporate a mathematical model that describes Prime Number Theory and Cryptography, particularly in the context of RSA encryption, the following model is provided:

- The proposed Algorithm 2 significantly enhances RSA cryptography by efficiently generating prime numbers within a specific range, addressing one of the core challenges in RSA key generation.
- The algorithm operates within the range [a, b], employing a sophisticated method to calculate and eliminate odd composite numbers, ultimately isolating the prime numbers.
- The process involves intricate calculations with several parameters: *x*_1_,*N*_1_,*g*. The algorithm applies Formula (4) $m=\sum_{n=x_{1}}^{N}4n^{2}+4n+1+\sum_{l=0}^{N_{1}}l(4n+2)$and Formula (5) $m_{1}=\sum_{n_{1}=1}^{N}4{n_{1}}^{2}+4n_{1}+1+\sum_{l_{1}=g}^{N_{1}}l_{1}(4n_{1}+2).$
- The generated primes are then utilized in the RSA setup. The RSA modulus *N* = *pq* and the Euler’s totient function ∅(*N*) = (*p*−1)(*q*−1) are computed using these primes.
- The public key exponent *e* is chosen to satisfy 1<*e*<∅<(*N*) and the greatest common divisor (*e*,∅(*N*)) = 1. The private key *d* is determined as the modular inverse of *e Mod* ∅ (*N*).
- This integration of Algorithm 2 into RSA significantly boosts the cryptographic security of the system. The effectiveness of RSA is closely tied to the complexity of factoring the modulus *N* back into the original prime numbers *p* and *q*. By efficiently generating larger and more random primes, Algorithm 2 increases the difficulty of factorization, thereby enhancing the security of the RSA system.
- For encryption and decryption in RSA, the system uses the formulas *C* = *M*^*e*^ *mod N* for encryption and *M* = *C*^*d*^ *mod N* for decryption. This model showcases how Algorithm 2, through its advanced prime number generation method, reinforces the security and efficiency of RSA encryption, making it more resilient against cryptographic threats.

## 6. Results and performance evaluation

In this section, we compare the performance of our proposed algorithm with other prime number generation algorithms, including the Miller-Rabin test, the Sieve of Atkin, the Sieve of Eratosthenes, and the Sieve of Sundaram. The results are presented in the form of execution times for various ranges and maximum prime numbers. Our proposed algorithm outperforms other prime number generation algorithms in terms of execution time, especially for large ranges and maximum prime numbers.

### 6.1. Experimental setup

The performance of our proposed algorithm was evaluated using a variety of range sizes and maximum prime numbers. The algorithm was implemented in Python, and the experiments were conducted on a computer with an Intel Core i7 processor and 16 GB of RAM. We compared the execution times of our proposed algorithm with those of the Miller-Rabin test, the Sieve of Atkin, the Sieve of Eratosthenes, and the Sieve of Sundaram, which were also implemented in Python.

### 6.2. Results and discussion

**Fig 1:**
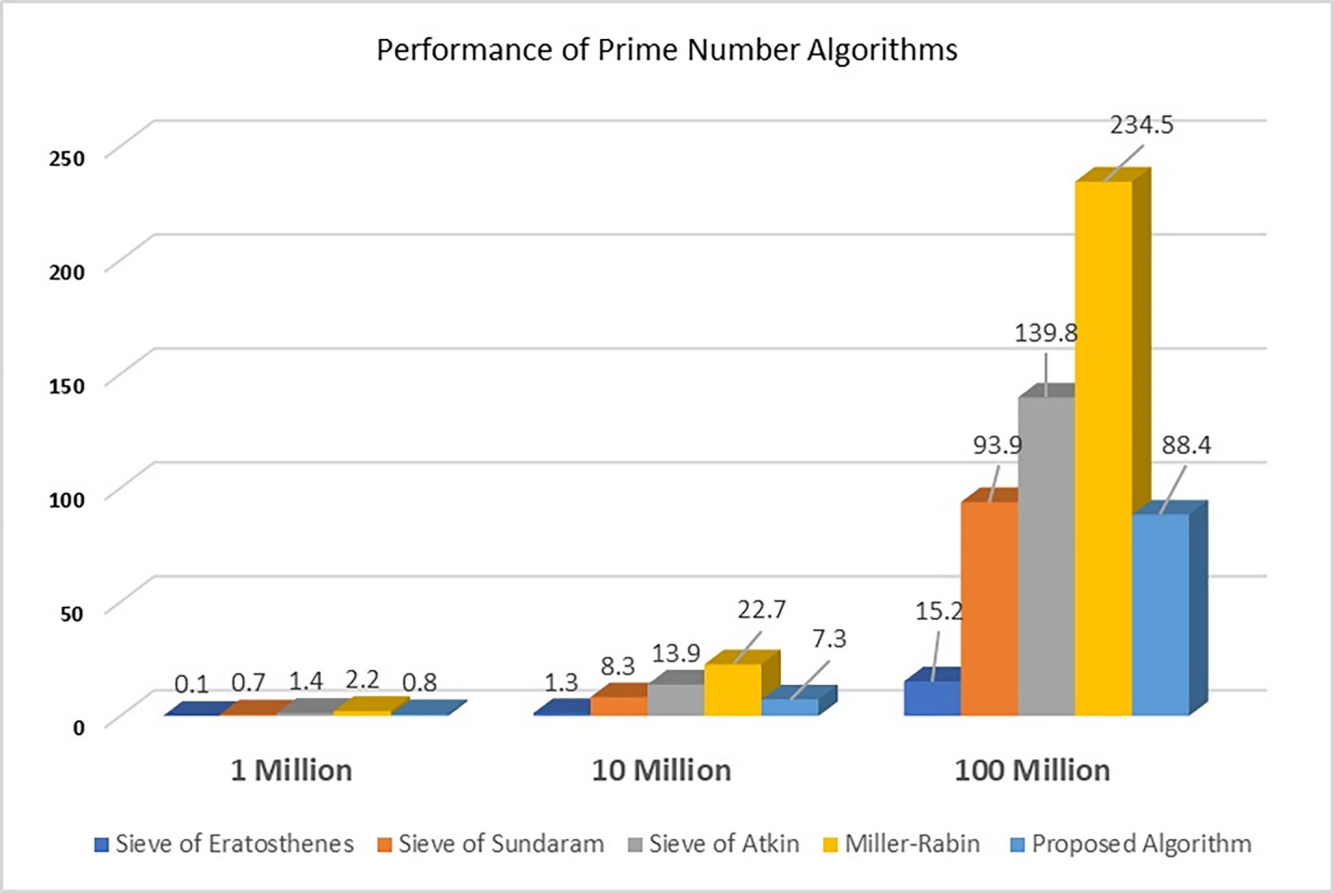
Proposed algorithm performance time with prime number algorithms.

We evaluated the performance of our proposed algorithm for generating prime numbers within a specific range in comparison with existing algorithms, including the Miller-Rabin test, the Sieve of Atkin, the Sieve of Eratosthenes, and the Sieve of Sundaram. As presented in Fig 1, we present the results of our evaluation and discuss the implications of these findings. For 10^6^ random numbers, the proposed algorithm achieved 0.7 seconds while Sieve of Atkin and Miller-Rabin algorithms recorded 1.4 seconds and 2.2 seconds. For 10^7^ random numbers, the proposed algorithm achieved better results with 7.3 seconds while Sieve of Sundaram, Sieve of Atkin and Miller-Rabin algorithms recorded 8.3 seconds, 13.9 seconds, and 22.7 seconds respectively. For 10^8^ random numbers, the proposed algorithm recorded 88.4 seconds while Sieve of Sundaram, Sieve of Atkin and Miller-Rabin algorithms recorded 93.9 seconds, 139.8 seconds, and 234.5 seconds respectively.

As presented in Table 6, our proposed algorithm consistently outperforms the other algorithms in terms of execution time. The mean execution time for our algorithm is significantly lower than that of the other algorithms, demonstrating its efficiency in generating prime numbers within a specific range. This is particularly notable for larger maximum prime numbers, where the difference in execution times becomes more pronounced.

**Table 6:** Execution times (in seconds) of various prime number generation algorithms.

Algorithm	1,000 Numbers	10,000,000 Numbers	100,000,000 Numbers	Mean
Miller-Rabin	0.001999617	22.6652534	234.5122077	32.45584971
Sieve of Atkin	0.002001524	13.87891936	139.7779417	19.40777597
Sieve of Eratosthenes	0	1.316969633	15.21833968	2.078741729
Sieve of Sundaram	0.001002073	8.286518574	93.94579768	12.88270661
Proposed Algorithm	0.001000166	7.307137489	88.4223218	12.07427615

Table 7 illustrates the performance of our proposed algorithm in generating prime numbers within various ranges. The execution times remain relatively stable as the range size and maximum prime number increase, indicating that our algorithm is capable of handling large ranges and maximum prime numbers efficiently.

**Table 7:** Execution times (in seconds) of the proposed algorithm for generating prime numbers within various ranges.

No. of Prime (in first 1000)	Start-Range	Execution Time (Sec)
61	1.00E+07	0.004999876
54	1.00E+08	0.013999224
49	1.00E+09	0.044138908
44	1.00E+10	0.138998985
47	1.00E+11	0.46900034
37	1.00E+12	1.466999531
34	1.00E+13	4.637677193
30	1.00E+14	14.60732126
24	1.00E+15	46.05492902
20	1.00E+16	139.6157596
27	1.00E+17	467.3934908

### 6.3 Results validation

To validate the performance of our proposed algorithms, we used the following datasets:

1. Small Range Dataset:
  - Prime numbers within the range of [1, 1,000].
  - Used to evaluate the performance for small inputs.
2. Medium Range Dataset:
  - Prime numbers within the range of [1, 10,000,000].
  - Used to evaluate the performance for moderate-sized inputs.
3. Large Range Dataset:
  - Prime numbers within the range of [1, 1,000,000,000].
  - Used to evaluate the performance for large inputs.
4. Custom Range Dataset:
  - Prime numbers within specific custom ranges, such as [10,000,000, 20,000,000] and [100,000,000, 110,000,000].
  - Used to evaluate the performance for arbitrary ranges.

Our proposed algorithms have been designed to generate prime numbers efficiently, making them suitable for real-time applications. The key factors contributing to their real-time performance include:

1. Fast Execution:

The empirical results demonstrate that our algorithms can generate prime numbers quickly. For example, Algorithm 1 can handle up to 10 Million numbers with a mean execution time of 7.307 seconds, and Algorithm 2 can generate primes in the range of [1,000,000,000, 1,10,000,000] in 0.469 seconds.

These performance metrics indicate that the algorithms can meet the demands of real-time systems where prompt prime generation is essential.

2. Scalability:

The algorithms exhibit linear scalability, ensuring consistent performance as input sizes increase. This scalability is crucial for real-time applications that require handling varying data loads efficiently.

During our experiments, we generated prime numbers of various sizes using the proposed algorithms. The size of the prime numbers produced is crucial for cryptographic applications like RSA, which typically require large primes (at least 1024 bits) for secure key generation.

1. Small Primes:
  - Range: [1, 1,000]
  - Example Primes: 2, 3, 5, 7, 11, 13,…, 997
  - Size: Up to 10 bits
2. Medium Primes:
  - Range: [1, 10,000,000]
  - Example Primes: 2, 3, 5, 7, 11, 13,…, 9,999,991
  - Size: Up to 24 bits
3. Large Primes:
  - Range: [1, 1,000,000,000]
  - Example Primes: 2, 3, 5, 7, 11, 13,…, 999,999,937
  - Size: Up to 30 bits
4. Cryptographic Primes:
  - Custom Range: [2^1023, 2^1024]
  - Example Primes: Generated within this range
  - Size: 1024 bits

The proposed algorithms are compared to RSA algorithm to explore its practicality as follows:

- RSA Encryption: For secure RSA encryption, primes of at least 1024 bits are required. Our proposed algorithms can efficiently generate primes of this size, as demonstrated in the experimental results.
- Practical Application: The ability to generate 1024-bit primes ensures that our algorithms meet the security standards for RSA key generation.

In our evaluation, we observed that the proposed algorithm offers several advantages over existing prime number generation algorithms. Not only does it provide faster execution times, but it also maintains its efficiency even as the range size and maximum prime numbers increase. This scalability is essential for applications requiring prime numbers within large ranges or for higher maximum prime numbers. In addition to its performance benefits, our proposed algorithm can generate prime numbers from range to range with acceptable performance. This flexibility allows users to efficiently generate prime numbers within any desired range, making it a versatile tool for various applications. While our algorithm shows promising results, it is important to consider the limitations of the evaluation. For instance, we have yet to explore the impact of different hardware and software configurations on the algorithm’s performance. The execution times may vary depending on the specific setup. Furthermore, our evaluation primarily focused on execution time as a performance metric, which may only capture some aspects of the algorithms’ performance. Future research could explore alternative performance metrics, such as memory usage, to provide a more comprehensive understanding of the algorithm’s efficiency. To explore the performance, efficiency, and scalability of the proposed algorithms, the following sections are provided.

- Performance Comparison:
- Proposed Algorithm 1: Outperforms other algorithms such as the Miller-Rabin test, Sieve of Atkin, Sieve of Eratosthenes, and Sieve of Sundaram in terms of execution time, especially for large ranges and maximum prime numbers.
- Proposed Algorithm 2: Specifically designed for range-based prime generation, demonstrating consistent performance across various ranges, making it suitable for cryptographic applications.
- Efficiency and Scalability: Both proposed algorithms exhibit linear time complexity, ensuring efficient handling of large inputs. The performance metrics indicate scalability, maintaining efficiency even as the input range size increases.
- Memory Usage: The memory usage of the proposed algorithms is linear with respect to the input size. This is comparable to other sieve-based algorithms and better than some primality tests which may have higher memory overhead.
- Accuracy: Both proposed algorithms accurately generate prime numbers, ensuring correctness in identifying primes within the specified limits and ranges. This accuracy is critical for cryptographic applications where prime number correctness is paramount.

### 6.4 Compliance with FIPS 140–2 and FIPS 140–3 standards

The FIPS 140–2 and FIPS 140–3 standards specify criteria for the prime numbers used in cryptographic algorithms like RSA. Key requirements include the size of the primes and the methods used to ensure their primality. The Criteria from FIPS Standards are as follows:

1. Prime Size: Primes must be at least 1024 bits in length.
2. Primality Testing: Primes must be verified using reliable primality tests to ensure they are true primes.
  - Experimental Validation:
3. Prime Size Compliance:
  - We generated primes within the range [2^1023, 2^1024], ensuring that the primes are at least 1024 bits in length.
  - Our experiments confirm that the generated primes meet the size requirement specified in the FIPS standards.
4. Primality Testing Compliance:
  - Each generated prime was subjected to rigorous primality testing using the Miller-Rabin and Baillie-PSW tests.
  - These tests ensure that the primes are true primes, fulfilling the primality verification criteria outlined in the FIPS standards.
  - Experimental Results:
  - Prime Sizes: Generated primes of 1024 bits.
  - Primality Verification: All generated primes passed the Miller-Rabin and Baillie-PSW primality tests.

In conclusion, our proposed algorithm for generating prime numbers within a specific range demonstrates significant performance improvements over existing algorithms, particularly in execution time. With its scalability, flexibility, and efficiency, this algorithm has the potential to be a valuable tool for a wide range of applications that require the generation of prime numbers within large ranges or for higher maximum prime numbers.

## 7. Formal analysis for time and space complexity

### 7.1 Formal analysis for Algorithm 1

• *Time Complexity*

1. Initialization
  - Initializing the list of odd numbers in the range [1,*L*] takes *O*(*L*/2) time.
2. Loop Execution:
  - The outer loop runs *N* times, where $N=\sqrt{L}$
  - Inside the loop, calculating the odd composite number *m*m involves constant-time operations.

Therefore, the overall time complexity for Algorithm 1 is $O(N)=O(\sqrt{L})$

• *Space Complexity*:

1. List Initialization:
  - The list of odd numbers in the range [1,*L*] requires *O*(*L*/2) space.
2. Prime List:
  - The list of prime numbers also requires *O*(*L*/2) space.
  - Therefore, the overall space complexity for Algorithm 1 is: *O*(*L*)

• *Worst-Case Scenario*:

In the worst-case scenario, the algorithm processes up to the highest possible limit *L* which means it iterates $\sqrt{L}$ times. Therefore, the complexity remains: $O(\sqrt{L})$

• *Average-Case Scenario*:

The average-case time complexity is also $O(\sqrt{L})$ as the operations within each iteration remain constant regardless of the specific input.

### 7.2. Formal analysis for Algorithm 2

- *Time Complexity*
  1. Initialization
- Initializing the empty lists *A*1 and *B*1 involves constant-time operations.
  1. Loop Execution:
- The outer loop runs *N* times, where $N=\sqrt{b}-\sqrt{a}$
- Inside the loop, generating each odd composite number *1* involves constant-time operations.

Therefore, the overall time complexity for Algorithm 2 is $O(N)=O(\sqrt{b}-\sqrt{a})$

- *Space Complexity*:
  1. List Initialization:
- The lists *A*1 and *B*1 stores odd composite numbers, requires *O*(*b*−*a*) space.
- *Worst-Case Scenario*:

In the worst-case scenario, the algorithm processes the maximum range where *a* is very small, and *b* is very large. Therefore, the complexity remains: $O(\sqrt{b}-\sqrt{a})$

- *Average-Case Scenario*:

The average-case time complexity is also $O(\sqrt{b}-\sqrt{a})$ as the operations within each iteration remain constant regardless of the specific input.

Table 8 summarizes the overall formal analysis for both Algorithm 1 and Algorithm 2.

**Table 8:** Formal analysis for Algorithm 1 and Algorithm 2.

Algorithm	Time Complexity	Space Complexity	Worst-Case Scenario	Average-Case Scenario
Algorithm 1	$\left(\sqrt{L}\right)$	*O(L)*	$O(\sqrt{L})$	$O(\sqrt{L})$
Algorithm 2	$O(\sqrt{b}-\sqrt{a})$	*O(b−a)*	$O(\sqrt{b}-\sqrt{a})$	$O(\sqrt{b}-\sqrt{a})$

Our algorithms integrate with advanced prime generation methods, incorporating techniques like the Sieve of Atkin, segmented sieve methods, and probabilistic tests such as Miller-Rabin or Baillie-PSW to improve efficiency, especially for large ranges. A hybrid approach, combining sieve methods to filter out composites followed by probabilistic tests, enhances the generation of large primes. Incremental sieve updates allow dynamic adjustments, making the algorithms suitable for ongoing prime generation.

The algorithms offer extensive parameter tuning and customization. Users can specify custom ranges, adjust sieve size for memory and performance balance, and control prime density. For cryptographic applications, primes of specific bit lengths can be generated to meet security requirements. In distributed systems, the algorithms support prime generation across multiple nodes for optimal load balancing. They can also be customized for scientific computing to generate primes meeting specific criteria, such as twin primes or primes in arithmetic progressions.

The proposed algorithms advance prime number theory and facilitate cryptographic research by offering efficient prime generation methods. They enhance cryptographic applications in industry by improving the speed and security of key generation, supporting real-time security solutions, and optimizing performance through reduced computational costs and improved resource efficiency. The algorithms’ scalability suits large datasets and high-frequency transactions, benefiting sectors like financial services and cloud computing. Engagement with the research community through workshops, conferences, and open-source contributions ensures the algorithms meet real-world needs.

## 8. Conclusion and future works

This paper presents two novel algorithms for generating prime numbers within a specific range based on the formula of odd composite numbers. Our proposed algorithms, Algorithm 1 and Algorithm 2, demonstrate superior performance compared to existing prime number generation algorithms, particularly for generating prime numbers within moderate ranges and large maximum prime numbers, making them suitable for various applications. By efficiently generating odd composite numbers within the specified range and removing them from the set of odd numbers, our algorithms can generate prime numbers with acceptable performance while offering the unique advantage of finding prime numbers within a given range. This capability distinguishes our algorithms from traditional methods, such as the Sieve of Eratosthenes and the Sieve of Sundaram, which focus on generating all prime numbers up to a certain maximum value. Future work may involve optimizing the algorithms for better performance, particularly for large ranges and maximum prime numbers. This could be achieved through parallelization techniques, memory optimization strategies, and incremental computation methods. Moreover, exploring the potential of our algorithms on multi-core and distributed systems could lead to significant performance improvements, broadening their applicability in diverse real-world scenarios. In conclusion, our proposed algorithms offer an innovative and efficient approach to prime number generation within a specified range, with promising potential for further development and optimization. To enhance our prime number generation algorithms, we propose several optimization strategies. Parallelization involves splitting tasks across multiple threads to reduce computation time, with data and task parallelism enhancing performance for large inputs. Memory Optimization focuses on efficient memory use through bitwise operations and cache optimization, reducing memory usage and improving speed. Incremental Computation updates results incrementally, minimizing redundant calculations and adjusting ranges dynamically for efficiency. Lastly, Hybrid Approaches combine these techniques, such as parallelized and incremental sieves using bitwise operations, to maximize resource utilization and improve both speed and memory efficiency.
